# Recombinant lipidated FLIPr effectively enhances mucosal and systemic immune responses for various vaccine types

**DOI:** 10.1038/s41541-023-00680-4

**Published:** 2023-06-02

**Authors:** Ming-Shu Hsieh, Mei-Yu Chen, Chia-Wei Hsu, Yu-Wen Tsai, Fang-Feng Chiu, Cheng-Lung Hsu, Chang-Ling Lin, Chiao-Chieh Wu, Ling-Ling Tu, Chen-Yi Chiang, Shih-Jen Liu, Ching-Len Liao, Hsin-Wei Chen

**Affiliations:** 1 National Institute of Infectious Diseases and Vaccinology, National Health Research Institutes, Miaoli, Taiwan; 2 Division of Hematology-Oncology, Department of Internal Medicine, Chang Gung Memorial Hospital, Chang Gung University, Taoyuan, Taiwan; 3 Graduate Institute of Biomedical Sciences, China Medical University, Taichung, Taiwan; 4 Graduate Institute of Medicine, Kaohsiung Medical University, Kaohsiung, Taiwan

**Keywords:** Adjuvants, Adjuvants

## Abstract

Formyl peptide receptor-like 1 inhibitor protein (FLIPr) is an immune evasion protein produced by *Staphylococcus aureus*, and FLIPr is a potential vaccine candidate for reducing *Staphylococcus aureus* virulence and biofilm formation. We produced recombinant lipidated FLIPr (rLF) to increase the immunogenicity of FLIPr and showed that rLF alone elicited potent anti-FLIPr antibody responses to overcome the FLIPr-mediated inhibition of phagocytosis. In addition, rLF has potent immunostimulatory properties. We demonstrated that rLF is an effective adjuvant. When an antigen is formulated with rLF, it can induce long-lasting antigen-specific immune responses and enhance mucosal and systemic antibody responses as well as broad-spectrum T-cell responses in mice. These findings support further exploration of rLF in the clinic as an adjuvant for various vaccine types with extra benefits to abolish FLIPr-mediated immunosuppressive effects.

## Introduction

*Staphylococcus aureus* can be an innocuous member of the microbiota. However, it can become an invasive pathogen when it has access to sterile tissues once epithelial barriers or immune systems become compromised^1–3^. Nasal *Staphylococcus aureus* carriage is the main reservoir for this species, which is a major risk factor in the pathogenic process of hospital- and community-acquired infections^4,5^. *Staphylococcus aureus* has developed several strategies to escape attack from the immune system of hosts. The strategies of this bacterium include resistance to antimicrobial peptides and inhibition of neutrophil recruitment, phagocytosis, and killing by ROS and neutrophils^6^. Therefore, products of surface and immune evasion genes are potential candidates for *Staphylococcus aureus* vaccine development^7^.

FLIPr is produced by *Staphylococcus aureus*, and it is an immune evasion protein that evades the immune system in at least two ways. One way is through FLIPr binding to C1q subcomponents or the C1q complex, which reduces classical complement pathway activation and increases *Staphylococcus aureus* survival in whole human blood^8^. The other way is FLIPr binding to various Fcγ receptor subclasses and the competitive blocking of IgG-ligand binding. These interactions inhibit Fc γ receptor-mediated effector functions, including opsonophagocytosis, the subsequent intracellular killing of *Staphylococcus aureus* by neutrophils and the Ab-dependent cellular cytotoxicity of tumor cells by both neutrophils and NK cells^9^. In addition, FLIPr was detected in a majority of the infecting isolates during biofilm formation in vitro^10^. Therefore, FLIPr is a potential target for vaccination to reduce *Staphylococcus aureus* virulence and biofilm formation.

Many infections begin on mucosal surfaces. Mucosal immunity is believed to be the first line of defense against a variety of pathogen invasions. Therefore, there is an urgent need to develop mucosal vaccines that trigger protective immunity in mucosal and systemic immune compartments^6,11–14^. Inactivated virus and recombinant protein (subunit vaccines) are noninfectious. These two vaccine types have significant safety advantages over live or live-attenuated vaccines. However, inactivated virus and recombinant protein have less immunogenicity than live or live-attenuated vaccines. In general, inactivated virus and recombinant protein need to be formulated with adjuvants to induce robust immune responses. Currently, mucosal adjuvants approved for human vaccines are rare.

Several studies have shown that bacterial-derived lipoproteins and synthetic lipopeptides can activate antigen-presenting cells through Toll-like receptor signaling pathways and then enhance humoral and cellular responses^15–18^. Based on these results, we have developed a novel technology to express recombinant lipoprotein using an *Escherichia coli*-based system for the development of subunit vaccines with high immunogenicity^19^. With this technology, we may acquire the advantage of subunit vaccines (safety) but no disadvantages (low immunogenicity). This concept has been successfully applied to several vaccine candidates, including therapeutic vaccines for cancer immunotherapy^20,21^ and preventive vaccines against viruses^22–27^ and bacteria^28^. Several reports have pointed that Toll-like receptor agonists are potent mucosal adjuvants^29–32^. In particular, intranasal immunization of mice with antigen containing synthetic lipopeptide (Toll-like receptor-2 agonist) enhance systemic and mucosal immune responses^33,34^. Therefore, we hypothesize that recombinant lipoprotein produced by this technology has adjuvant properties like the synthetic lipopeptides.

In the present study, we prepared recombinant lipidated FLIPr (rLF) and evaluated its immunogenicity. We demonstrated that rLF alone elicited potent anti-FLIPr antibody responses to overcome FLIPr-mediated inhibition of phagocytosis. Importantly, we found that simply mixing rLF with inactivated virus or recombinant protein could enhance mucosal and systemic immune responses to inactivated virus or recombinant protein. These results provide important information for future clinical studies on rLF as an adjuvant and a vaccine candidate to block the immune evasion function of FLIPr.

## Results

### Recombinant lipidated FLIPr alone induces potent antibodies to control FLIPr-mediated inhibition of phagocytosis successfully

**Fig. 1:**
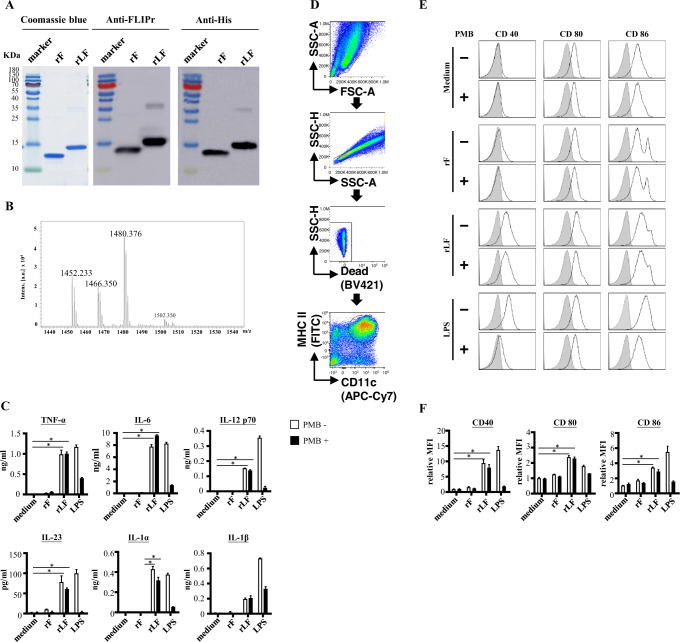
Production and characterization of rF and rLF. Purified rF and rLF were examined by 10% reducing SDS-PAGE followed by (**A**) Coomassie Blue staining and immunoblotting with anti-FLIPr or anti-HisTaq antibodies. **B** The N-terminal fragments of rLF were obtained and identified after trypsin digestion of rLF. The digested sample was analyzed on a Waters® MALDI micro MX™ mass spectrometer. The MALDI-TOF MS spectra revealed the presence of three major peaks with m/z values of 1452, 1466, and 1480. The activation of bone marrow-derived dendritic cells induced by rLF. Mouse bone-marrow-derived dendritic cells were cultured in medium supplemented with rF or rLF (5 µg/mL) in the presence or absence of polymyxin B (PMB, 25 µg/mL). Dendritic cells stimulated with medium alone or lipopolysaccharide (LPS) were used as controls. **C** The supernatants were collected at 20 h after stimulation. The levels of TNF-α, IL-6, IL-12p70, IL-23p19, IL-1α, and IL-1β were determined using ELISA kits. The cells were harvested and analyzed by flow cytometry. **D** The gating strategy for the bone-marrow-derived dendritic cells is shown. Dead cells were excluded from analysis by staining with a Live/Dead® fixable dead cell stain dye. Bone marrow-derived dendritic cells gated on live/ CD11c^+^ MHCII^+^ cells. **E** The expression levels of CD40, CD80, and CD86 on a CD11c^+^ MHCII^+^ gated population were analyzed by flow cytometry. A representative experiment is shown. **F** The mean fluorescence intensity for cells stimulated with medium alone was defined as the basal expression level. The relative mean fluorescence intensities (the fold change compared to the medium control) were plotted. The data represent the means ± SD of the mean from three independent experiments. The statistical significance was determined using the Kruskal-Wallis test with Dunn’s multiple comparison test. *p < 0.05.

Recombinant FLIPr (rF) and rLF were produced using *E. coli*-expressing systems. Both purified proteins were analyzed by 10% SDS-PAGE, followed by staining with Coomassie Blue or subject to further examination by immunoblotting with anti-FLIPr or anti-His antibodies (Fig. 1A, raw images in the Supplementary Fig. 1). To examine the lipidation of rLF, the mass of the N-terminal fragment of rLF was measured. Three major peaks with m/z values of ~1452, ~1466, and ~1480 were identified in the MALDI-TOF MS spectra (Fig. 1B). These peaks represent lipid-CSQEAK N-terminus fragment of lipoprotein^19^. There are no such peaks in the non-lipidated protein. Therefore, these peaks are considered as the lipidation signature which can be identified in other recombinant lipoproteins^20,21,23–25,27,28,35^ produced by the same technology^19^. These results suggest that rLF is lipidated at its N-terminus.

We examined the capacity of rLF to activate innate immune cells in vitro using bone marrow-derived dendritic cells (BMDCs). To exclude the effect of little residual endotoxin during recombinant protein preparation, polymyxin B was added to the rF or rLF in parallel experiments. rLF was able to stimulate the production of TNF-α, IL-6, IL-12p70, IL-23p19, IL-1α, and IL-1β by BMDCs. Cytokine production was not abrogated in the presence of polymyxin B. In contrast, rF (without lipidation) did not stimulate cytokine production (Fig. 1C). Next, the expression levels of CD40 and costimulatory molecules (CD80 and CD86) on BMDCs upon stimulation with rF or rLF were analyzed by flow cytometry. The gating strategy is shown in Fig. 1D. As shown in Fig. 1E, rLF stimulation increased the expression of CD40, CD80, and CD86, while rF (without lipidation) stimulation was ineffective at increasing the expression of these molecules. The expression of CD40, CD80, and CD86 on BMDCs was dramatically reduced upon LPS stimulation in the presence of polymyxin B. In contrast, the addition of polymyxin B did not affect the ability of rF and rLF to activate BMDCs. When BMDCs were stimulated with medium alone, the mean fluorescence intensities (MFIs) of CD40, CD80, or CD86 on BMDCs were considered the basal expression levels. The relative MFIs (the fold change compared to the medium control) from 3 independent experiments are summarized in Fig. 1F. These results indicate that rLF functions by activating BMDCs.

**Fig. 2:**
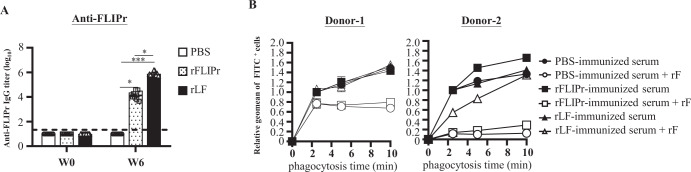
rLF-immunized sera overcome the FLIPr-mediated inhibition of phagocytosis by human neutrophils. **A** Antibody responses induced by rLF. Groups of C57BL/6 mice (*n* = 9/group) were intranasally immunized three times with PBS, rF or rLF at a 2-week interval. PBS-immunized mice served as the control. The titers of anti-FLIPr IgG antibodies in the sera before immunization (W0) or 6 weeks after immunization (W6) were determined by ELISA. Statistical significance was determined using the Kruskal-Wallis test with Dunn’s multiple comparison test. *p < 0.05; **p < 0.01; and ***p < 0.001. **B** The sera from immunized mice were collected at 6 weeks post-immunization and used to opsonize the phagocytosis of fluorescent staphylococci by human neutrophils. The presence of 30 µg/ml rF was used to inhibit phagocytosis. Phagocytosis was determined as the relative geometric mean fluorescence intensity of cells with fluorescent bacteria. The data represent the means ± SD from two independent experiments.

To examine whether rLF is superior to rF in eliciting functional immunities, groups of C57BL/6 mice were immunized with PBS, rF or rLF three times with a two-week interval. FLIPr-specific antibody titers in mice immunized with rLF were significantly higher than those in mice immunized with rF or PBS (Fig. 2A). Next, we performed phagocytosis experiments to test whether anti-FLIPr sera could break down the FLIPr-mediated inhibition of phagocytosis. Fluorescently labeled staphylococci were opsonized with vaccinated sera. Human neutrophils were incubated with or without rF (30 µg/mL), and phagocytosis was determined at increasing times. In the presence of rF, phagocytosis was inhibited. The inhibitory effects of rF on phagocytosis were abolished when rLF-vaccinated sera but not rF- or PBS-vaccinated sera were added (Fig. 2B). These results indicate that rLF is superior to its non-lipidated counterpart in inducing antibody responses and further overcoming FLIPr-mediated immunosuppressive effects.

### Recombinant lipidated FLIPr is a potent adjuvant simply mixed with antigen to promote robust immunity

**Fig. 3:**
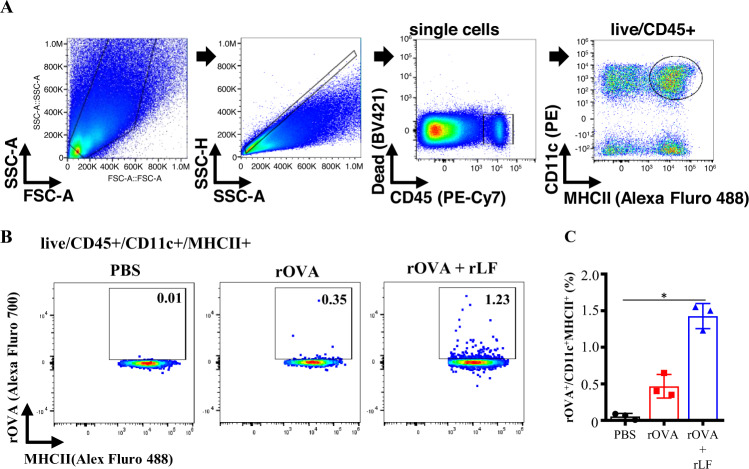
Formulation of rOVA with rLF efficiently enhances delivery of rOVA to NALT dendritic cells. A group of C57BL/6 mice was intranasally administered 30 μg of Alexa Fluor 700-labeled rOVA alone or adjuvanted with 10 μg of rLF. Mice administered PBS alone were used as controls. **A** The gating strategy for the dendritic cell population in NALT cells was performed 18 h after administration. A single-cell suspension of NALT cells (pooled from 3 mice) was obtained by mechanical disruption and collagenase digestion of nasal tissue. Dead cells were excluded from analysis by staining with a Live/Dead^®^ fixable dead cell stain dye. DCs from the NALT gated on live/CD45^+^/ CD11c^+^ MHCII^+^ cells. **B** One representative experiment of three individual experiments showed the frequencies of rOVA-labeled CD11c^+^ MHCII^+^ cells. **C** Cumulative data from three individual experiments are summarized. The data are presented as the means ± SEM (*n* = 3). Statistical significance was determined using the Kruskal-Wallis test with Dunn’s multiple comparison test. *p < 0.05.

Having shown that rLF alone promotes the activation of BMDCs and the induction of FLIPr-specific immune responses, we wondered whether rLF has adjuvant effects when formulated with antigens. Recombinant ovalbumin (rOVA) was used as a model antigen. First, we evaluated whether antigen formulated with rLF increases the efficacy of antigen delivered to antigen-presenting cells. Groups of mice were intranasally administered PBS or Alexa Fluor 700-conjugated rOVA alone or mixed with rLF. The frequencies of rOVA-harboring CD11c^+^ MICH II^+^ cells in NALT (pooled from 3 mice) were examined by flow cytometry 18 h after administration. The gating strategy is shown in Fig. 3A. The frequencies of rOVA-harboring CD11c^+^ MICH II^+^ cells increased in mice administered rOVA with rLF (Fig. 3B). The results from 3 independent experiments are summarized in Fig. 3C. These results show that rOVA formulated with rLF via intranasal administration can increase the frequencies of rOVA-harboring dendritic cells in NALT.

**Fig. 4:**
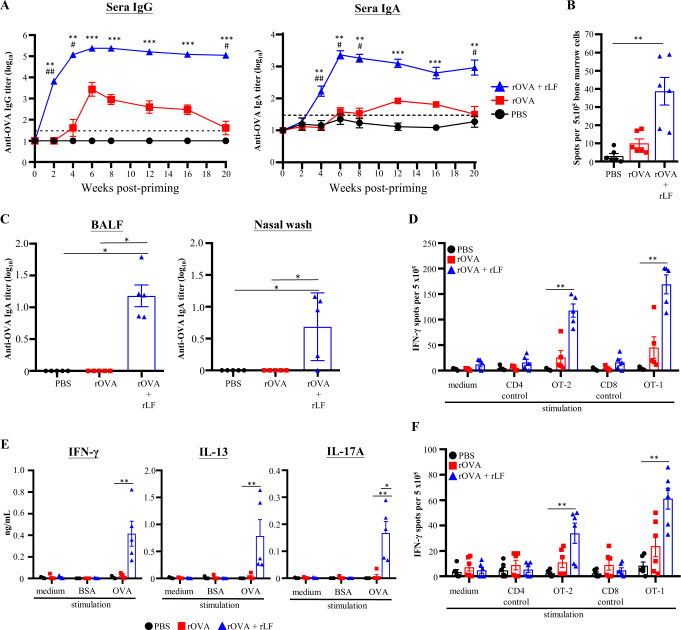
Antibody responses and T-cell responses induced by intranasal administration of rOVA-adjuvanted rLF. Groups of C57BL/6 mice (*n* = 5 or 6/group) were intranasally immunized three times with 30 µg of rOVA or rOVA mixed with 10 µg of rLF at 2-week intervals. Mice immunized with PBS alone (without antigens) served as controls. **A** Sera were collected at the indicated time points. OVA-specific IgG and IgA titers were assessed by ELISA. The detection limit is indicated by the dotted line on the y-axis, which indicates the initial dilution factor of the sample. Statistical significance was determined using the Kruskal-Wallis test with Dunn’s multiple comparison test. **p < 0.01; ***p < 0.001 vs. PBS. #*p* < 0.05; ##*p* < 0.01 vs. rOVA. **C** Samples of BALF and nasal wash were collected from mice 6 weeks after the first vaccination. The reactivity of OVA-specific IgA antibody titers in BALF and nasal wash was assessed by ELISA. **D** Splenocytes were harvested 6 weeks after the first vaccination. Cells were cultured and stimulated with OT-2, OT-1, control peptides, or medium alone for 3 days in an anti-INF-γ-coated 96-well ELISPOT plate. IFN-γ responses were measured using an ELISPOT assay and are expressed as spot-forming units per 5 × 10^5^ cells. **E** Splenocytes were cultured and stimulated with rOVA for 3 days. Cells stimulated with BSA or medium alone served as controls. The supernatants were collected to evaluate the levels of IFN-γ, IL-13 and IL-17A by ELISA. Data represent the means ± SE of the mean. The results shown here are from one of two representative experiments. **B** OVA-specific antibody-secreting cells in bone marrow and (**F**) IFN-γ producing cells in the spleen were evaluated using ELISPOT at 20 weeks after the first vaccination. Data represent the means ± SE of the mean. Statistical significance was determined using the Kruskal-Wallis test with Dunn’s multiple comparison test. *p < 0.05; **p < 0.01; and ***p < 0.001.

Next, we investigated the immune responses induced by various formulations. Groups of C57BL/6 mice were intranasally treated three times at 2-week intervals with 30 µg of rOVA or rOVA adjuvanted with rLF. Mice given PBS alone (without antigen) served as controls. Anti-OVA IgG antibodies were quickly generated in mice at 2 weeks after immunization with one dose of rOVA plus rLF. Anti-OVA IgG antibody titers were further elevated following boosting and maintained for at least 20 weeks after the initial priming (Fig. 4A). In parallel, substantial anti-OVA IgA antibodies were elicited in mice immunized with rOVA plus rLF. These anti-OVA IgA antibodies were also maintained for at least 20 weeks after the initial priming. In contrast, detectable anti-OVA IgG antibody titers were elicited in mice immunized with rOVA alone at 4 weeks post-priming (two doses). In addition, the anti-OVA IgG antibody titers in these mice peaked at 6 weeks post-priming and gradually declined. Consistent with the anti-OVA antibody titers in the sera, antibody-secreting cells in the bone marrow of mice immunized with rOVA plus rLF were still present 20 weeks post-priming and were higher than those in the bone marrow of mice immunized with PBS or rOVA (Fig. 4B). These results suggest that rLF has adjuvant effects and that vaccination with rOVA plus rLF induces long-term systemic antibody responses.

We further examined the anti-OVA IgA titers in bronchoalveolar lavage fluid (BALF) and nasal wash (NW) 6 weeks post-priming. Anti-OVA IgA antibodies were present in the BALF and NW obtained from mice immunized with rOVA plus rLF. No detectable anti-OVA IgA antibodies in the BALF or NW were obtained from mice immunized with PBS or rOVA alone (Fig. 4C). These results indicate that vaccination with rOVA with rLF elicits strong systemic and mucosal antibody responses.

We then examined the T-cell responses elicited by intranasal vaccination of C57BL/6 mice with rOVA or rOVA plus rLF. Mice immunized with PBS alone served as negative controls. One week after the last immunization, the frequencies of IFN-γ-producing cells in the spleens were evaluated by ELISPOT. For all the splenocytes, the frequencies of IFN-γ-producing cells were at background levels when there was no stimulation (medium) or stimulation with control peptides. Notably, the splenocytes in mice immunized with rOVA plus rLF generated higher frequencies of IFN-γ-producing cells than those in mice immunized with PBS or rOVA after stimulation with OT-2 (a CD4 epitope) or OT-1 (a CD8 epitope) peptides (Fig. 4D). These results show that vaccination with rOVA plus rLF is capable of inducing OVA-specific CD4^+^ and CD8^+^ T-cell responses.

To evaluate the T helper cell profiles, the levels of IFN-γ (Th1-associated cytokines), IL-13 (Th2-associated cytokines), and IL-17A (Th17-associated cytokines) produced by the splenocytes of vaccine-immunized mice were examined. Supernatants obtained from all of the splenocytes secreted low or barely detectable levels of IFN-γ, IL-13, and IL-17A without stimulation (medium) or stimulation with bovine serum albumin (BSA). After stimulation with rOVA, the levels of IFN-γ, IL-13, and IL-17A in supernatants obtained from mice immunized with rOVA plus rLF were higher than those obtained from mice immunized with PBS or rOVA (Fig. 4E). These results suggest that vaccination with rOVA plus rLF triggers a broad spectrum of T helper cell responses.

To ascertain whether the T-cell responses were persistent, similar to the antibody responses, we examined the recall activity of antigen-specific T cells on week 20 after priming. Consistently, high frequencies of IFN-γ-producing cells were still present after stimulation with OT-2 or OT-1 peptides (Fig. 4F). These results suggest that mice immunized with rOVA plus rLF can elicit long-lived memory T-cell responses.

### Preexisting anti-FLIPr antibody does not abolish the adjuvant activity of rLF

**Fig. 5:**
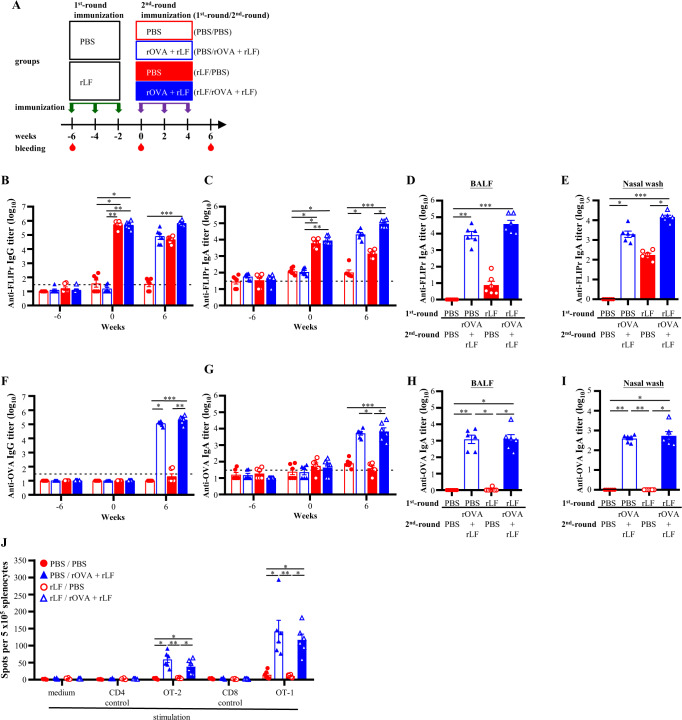
Preexisting anti-FLIPr antibodies do not diminish the antibody responses and T-cell responses induced by intranasal administration of rOVA adjuvanted with rLF. The experimental flow chart is shown (**A**). Groups of C57BL/6 mice (*n* = 12/group) were immunized with PBS or rLF three times at a two-week interval by intranasal administration. The mice in each group were randomly divided into 2 subgroups (*n* = 6/group) and treated with PBS or rOVA adjuvanted with rLF at 6 weeks after priming. The sera were collected at week -6 (baseline), 0 (after the three immunizations with PBS or rLF) and 6 (after the three immunizations with PBS or rOVA adjuvanted with rLF). The FLIPr-specific IgG (**B**) and IgA (**C**) antibody titers in the serum are shown. The OVA-specific IgG (**F**) and IgA (**G**) antibody titers of serum are shown. The BALF and nasal wash were collected from mice on week 6 (after the three immunizations with PBS or rOVA adjuvanted with rLF). The FLIPr-specific IgA antibody titers of BALF (**D**) and nasal wash (**E**) were shown. The OVA-specific IgA antibody titers of BALF (**H**) and nasal wash (**I**) are shown. The detection limit is indicated by the dotted line on the y-axis, which indicates the initial dilution factor of the sample. **J** Splenocytes were harvested on week 6 (after the three immunizations with PBS or rOVA adjuvanted with rLF). Cells were cultured and stimulated with OT-2, OT-1, control peptides, or medium alone for 3 days in an anti-INF-γ-coated 96-well ELISPOT plate. IFN-γ responses were measured using an ELISPOT assay and are expressed as spot-forming units per 5 × 10^5^ cells. All data represent the means ± SE of the mean. Statistical significance was determined using the Kruskal-Wallis test with Dunn’s multiple comparison test. *p < 0.05; **p < 0.01; and ***p < 0.001. The detection limit is indicated by the dotted line on the y-axis, which indicates the initial dilution factor of the sample.

To assess whether the preexisting anti-FLIPr antibodies influenced the adjuvant function of rLF, groups of mice were immunized with PBS or rLF three times with a two-week interval during the first-round vaccination (week −6, −4, and −2). Both groups of mice were randomly divided into two subgroups (week 0) and then immunized with PBS or rOVA plus rLF (week 0, 2, and 4). The experimental scheme is shown in Fig. 5A. We first monitored anti-FLIPr antibodies over a time course of two rounds of immunization. The anti-FLIPr IgG (Fig. 5B) and IgA (Fig. 5C) antibodies in sera were induced after the first-round immunization with rLF but not after PBS immunization (week 0). After the second-round immunization (week 6), anti-FLIPr antibodies were detected in serum (Fig. 5B, C) and mucosa (Fig. 5D, E) samples which had been previously received vaccination containing rLF (PBS/rOVA + rLF, rLF/PBS, rLF/rOVA + rLF). In addition, mice received vaccination all containing rLF (rLF/rOVA + rLF) induced the highest anti-FLIPr antibody responses than other vaccination groups. Before the second-round immunization (week 0), the anti-FLIPr antibody titers were equivalent between the subgroups of the rOVA plus rLF- and PBS-immunized mice. The anti-OVA IgG (Fig. 5F) and IgA (Fig. 5G) antibody titers in sera and IgA antibody titers in BALF (Fig. 5H) and nasal wash (Fig. 5I) in the rOVA plus rLF-immunized mice with preexisting anti-FLIPr antibodies were successfully elicited and were comparable to those in mice without preexisting anti-FLIPr antibodies

Next, the T-cell responses were further examined. Similar to the antibody responses, IFN-γ-secreting cells were successfully elicited in mice with preexisting anti-FLIPr antibodies. Although the frequencies of IFN-γ-secreting cells following OT-2 or OT-1 stimulation in mice with preexisting anti-FLIPr antibodies were slightly lower than those in mice without preexisting anti-FLIPr antibodies but not statistically significant (Fig. 5J). These results suggest that preexisting anti-FLIPr antibodies do not destroy the adjuvant functions of rLF.

### Recombinant lipidated FLIPr acts as an adjuvant for subunit and inactivated vaccines

**Fig. 6:**
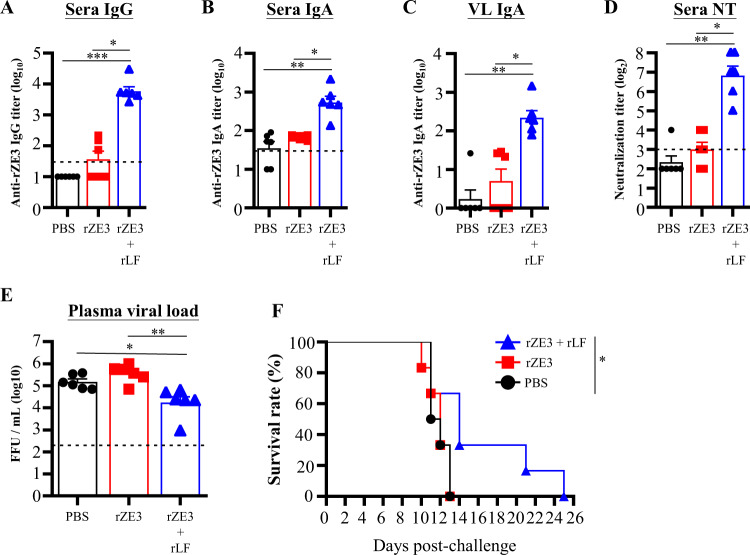
Antibody responses and protective effects induced by intranasal administration of rZE3 adjuvanted with rLF. Immunodeficient AG129 mice (*n* = 6/group) were vaccinated three times with PBS, rZE3, or rZE3 plus rLF via the intranasal route at two-week intervals. Sera were collected from vaccinated mice 6 weeks after the first vaccination. Sera and vaginal lavage (VL) samples were collected 6 weeks after the first vaccination. The titers of anti-rZE3 IgG (**A**) and IgA (**B**) antibodies in the sera and VL IgA (**C**) were determined by ELISA. Neutralization titers (**D**) in sera were determined by FRNT. The neutralizing antibody titer was defined as the reciprocal of the highest dilution that resulted in a 50% reduction in FFUs compared to the FFUs of control samples containing the virus alone. Animals were intraperitoneally injected with 80 FFUs of Zika virus (PRVABC59) in 0.2 mL of PBS at 6 weeks after the first administration. Plasma samples were collected 3 days after virus challenge. The viral titers (**E**) were evaluated by focus-forming assays. The viral titers were logarithmically transformed before statistical analyses. The data represent the means ± SE. The detection limit is indicated by the dotted line on the y-axis, which indicates the initial dilution factor of the sample. Statistical significance was determined using the Kruskal-Wallis test with Dunn’s multiple comparison test. *p < 0.05; **p < 0.01; and ***p < 0.001. **F** The overall survival of the mice is shown. Survival analysis was calculated by the log-rank test. *p < 0.05.

In view of the powerful adjuvant activity of rLF, we next evaluated the applicability of rLF formulated with a Zika subunit vaccine candidate. Groups of AG129 mice were intranasally treated three times at 2-week intervals with 30 µg of rZE3 or rZE3 adjuvanted with rLF. Mice administered PBS alone (without antigen) served as controls. Mice immunized with rZE3 plus rLF exhibited remarkable rZE3-specific IgG (Fig. 6A) and IgA (Fig. 6B) antibodies in their sera and IgA (Fig. 6C) antibodies in vaginal lavage 6 weeks after priming. There were few or barely detectable rZE3-specific IgG and IgA antibodies in mice immunized with rZE3 alone. Significantly, neutralizing antibodies were elicited only in the mice immunized with rZE3 plus rLF (Fig. 6D). These results indicate that mice intranasally immunized with rZE3 plus rLF induce IgG antibodies with neutralizing capacity and mucosal IgA antibodies.

To investigate the protective capacity induced by rZE3 plus rLF, the mice were challenged with Zika virus 6 weeks after priming. The viral loads in the plasma samples from rZE3 plus rLF-immunized mice were lower than those in rZE3- or PBS-immunized mice (Fig. 6E). Consistent with these findings, the rZE3 plus rLF-immunized mice had prolonged survival times in comparison with the PBS- or rZE3-immunized mice (Fig. 6F). These results suggest that rZE3 plus rLF induces protective immune responses in mice.

**Fig. 7:**
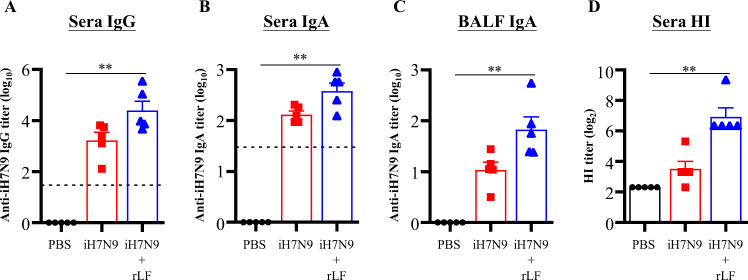
Antibody responses induced by intranasal immunization of iH7N9 adjuvanted with rLF. Groups of mice (*n* = 5/group) were vaccinated three times with PBS, iH7N9, or iH7N9 adjuvanted with rLF with a two-week interval by intranasal administration. Sera and BALF samples were collected 6 weeks after the first vaccination. The titers of anti-iH7N9 IgG (**A**) and IgA (**B**) antibodies in the sera and BALF IgA (**C**) were determined by ELISA. Vaccine-induced neutralizing antibody in sera against H7N9 was evaluated by hemagglutination inhibition assays (**D**). The detection limit is indicated by the dotted line on the y-axis, which indicates the initial dilution factor of the sample. Statistical significance was determined using the Kruskal-Wallis test with Dunn’s multiple comparison test. *p < 0.05; **p < 0.01.

Next, we evaluated the applicability of rLF formulated with inactivated H7N9 virus (iH7N9). Groups of BALB/c mice were intranasally administered iH7N9 or iH7N9 formulated with rLF three times at 2-week intervals. When the mice were immunized with iH7N9 plus rLF, iH7N9-specific IgG (Fig. 7A) and IgA (Fig. 7B) antibodies in sera and IgA (Fig. 7C) antibodies in BALF were elevated. Consistently, iH7N9 plus rLF-immunized mice induced hemagglutination inhibition (HI) titers superior to those of iH7N9- and PBS-immunized mice (Fig. 7D). These results suggest that rLF can enhance iH7N9-induced immune responses.

## Discussion

In this study, we report that rLF expressed from *E. coli* can stimulate innate immune responses that trigger the induction of functional antibodies against FLIPr to defeat FLIPr-mediated immunosuppressive effects (Fig. 2). rLF contains unique N-terminal lipidation, characteristic of signature peaks of recombinant lipidated proteins (Fig. 1B), which have been identified in other recombinant lipidated proteins^20–28^ produced by the same platform technology^19^. Recombinant lipoproteins produced from *E. coli*-expressed systems have been shown to activate antigen-presenting cells by triggering toll-like receptors^15–18^. We demonstrate that rLF has potent immunostimulatory activity, driving BMDC maturation and cytokine production. In contrast, rF did not have these functions (Fig. 1C–F). These results indicate that the lipid moiety of rLF is critical in BMDC activation. The characteristics of rLF lead the rLF itself to possess intrinsic adjuvant properties when mice immunized with rLF alone can generate stronger anti-FLIPr immunities than mice immunized with rF alone (Fig. 2).

Toll-like receptor agonists are not only to utilize as vaccine adjuvants^36^ but also as potential mediators of trained immunity^37^. Trained immunity is an immunological process, which describing the functional changes of innate immune cells and reprogramming of bone marrow progenitor cells lead to enhance immune responses against a secondary challenge after encountering a primary stimulation^38^. These results imply that rLF may also induce trained immunity to mediate better protection in our Zika virus challenge experiment (Fig. 6F). We will design experiments to test this hypothesis in our future studies.

DCs are professional antigen-presenting cells. They capture and process antigens that are critical to triggering CD4^+^ and CD8^+^ T-cell responses and then further regulate humoral immune responses^39–41^. *E. coli*-produced recombinant lipoproteins have been shown to improve the capture efficiency by DCs in vivo^21^. In this study, we show that there are bystander effects. When rOVA was formulated with rLF, it also increased the rOVA captured by DCs (Fig. 3). Since rLF is capable of stimulating DC maturation and increasing antigen uptake by DCs, we suggest that rLF may also be a potent adjuvant to drive the induction of vaccine-induced adaptive immune responses. This concept can be supported by four outcomes that enhance antigen-specific immune responses via the formulation of antigens with rLF.

The first is the enhancement of antibody responses. Formulating the antigen with rLF enhanced antigen-specific IgG and IgA titers in sera (Figs. 4A, 6A, B, 7A, B). These antibodies are functional, with neutralizing and HI capacities, as demonstrated by a Zika subunit vaccine (Fig. 6D) and an iH7N9 vaccine (Fig. 7D), respectively.

The second is the promotion of mucosal immune responses. Inducing antigen-specific IgA is a key consideration in terms of immune relevance after immunization with a mucosal vaccine. Formulating antigen with rLF can promote antigen-specific IgA titers not only in sera (Figs. 4A, 6B and 7B) but also in BALF and NW (Figs. 4C and 7C). Both IgA and IgG antibodies are important for conferring protective immunities in mice infected with influenza virus^42^. Plasma IgG antibodies serve as the backup for IgA-mediated protection in the nasal compartment, and IgG antibodies are the dominant antibodies in protecting the lungs. Formulating iH7N9 with rLF can elevate both H7N9-specific IgG and IgA (Fig. 7A–C), which benefits the host against H7N9 virus. Furthermore, intranasally administered rZE3 plus rLF elicited IgA antibodies in VL (Fig. 6C). These results suggest that rLF can promote systemic mucosal immune responses. Zika virus RNA has been detected in infected patients, in the semen, vagina, and cervix^43–45^. Therefore, the induction of mucosal immunity should be considered for novel Zika vaccine development. These results suggest that rLF is suitable for the above vaccines.

The third is the augmentation of T-cell responses. There are two major subsets of T cells, CD4^+^ and CD8^+^ T cells. In general, CD4^+^ T cells are designated as T helper cells that produce cytokines to regulate immune responses and orchestrate antibody production by B cells. CD8^+^ T cells are also called cytotoxic T lymphocytes (CTLs) that destroy virus-infected cells or transformed cancer cells. We have shown that the formulation of rOVA with rLF can augment the activation of CD4^+^ and CD8^+^ T cells (Fig. 4D) and stimulation of wide-range cytokine production profiles (Fig. 4E). These results indicate that rLF has adjuvant effects on both CD4^+^ and CD8^+^ T cells.

The fourth is the induction of memory and long-lasting immune responses. High levels of antigen-specific IgG and IgA were present in sera (Fig. 4A), and high frequencies of antigen-specific antibody-secreting cells in bone marrow (Fig. 4B), CD4^+^ T cells, and CD8^+^ T cells in the spleen (Fig. 4F) were recalled in mice immunized with rOVA plus rLF at 20 weeks after priming. These results represent memory, and long-lasting immune responses are elicited when antigens are formulated with rLF.

We demonstrate that mice immunized with rOVA plus rLF can enhance anti-OVA immune responses. It is interesting to know whether mice simultaneously generate anti-FLIPr immune responses when mice are immunized with rOVA plus rLF. Anti-FLIPr IgG and IgA antibodies were quickly generated in mice at 2 weeks after immunization with one dose of rOVA plus rLF. FLIPr-specific IgG and IgA antibody titers were further increased following boosting and maintained for at least 20 weeks after the initial priming (Supplementary Fig. 2A). In addition, anti-FLIPr IgA antibodies were only present in the BALF and NW obtained from mice immunized with rOVA plus rLF 6 weeks post-priming (Supplementary Fig. 2B). These results indicate that vaccination with rOVA plus rLF elicits strong OVA- (Fig. 4E) and FLIPr-specific (Supplementary Fig. 2A and 2B) systemic and mucosal antibody responses. We further examined the T helper cell profiles. After stimulation with rFLIPr, only splenocytes from mice immunized with rOVA plus rLF produced high levels of IFN-γ, IL-13, and IL-17A (Supplementary Fig. 2C and 2D). These results suggest that vaccination with rOVA plus rLF triggers a broad spectrum of OVA- (Fig. 4D, E) and FLIPr-specific (Supplementary Fig. 2C and 2D) T helper cell responses. Altogether, anti-FLIPr immune response profiles are similar to anti-OVA immune response profiles when mice immunized with rOVA plus rLF. These results support that rLF is not only a high immunogenic antigen but also an adjuvant.

A high percentage of the human population is colonized by *Staphylococcus aureus*^1–3^. In addition, mean IgG levels against FLIPr are significantly higher in bacteremia patients than in controls^10^. It is likely that many people have existing anti-FLIPr antibodies. The preexisting immunity against vaccine vectors in the host may damage the subsequent immune response to a vectored antigen^46,47^. Therefore, it is possible that preexisting anti-FLIPr antibodies can harm the adjuvant capacity of rLF. However, it did not destroy the adjuvant capacity of rLF in the mice with preexisting anti-FLIPr antibodies (Fig. 5). These results imply that rLF-adjuvanted vaccines can be used to immunize hosts, even those who have anti-FLIPr antibodies induced by infection or colonization with *Staphylococcus aureus*. Additionally, the rLF adjuvant can be repeatedly used in different vaccines regardless of whether the hosts have anti-FLIPr antibodies.

The safety issue is a key consideration for any new vaccine adjuvant. CpG is included in the safety assessment for comparisons. Body weights of mice are slightly reduced one day after each administration with rLF or CpG. The lost body weights are restored three days after administration and comparable to mice administration with PBS (Supplementary Fig. 3B). In terms of serum biochemical (Supplementary Fig. 3C), histopathologic (Supplementary Fig. 3D), and hematological (Supplementary Table 1) analyses, there is no difference among the mice administration with PBS, rLF, or CpG. Results in these preliminary safety assessments indicate that rLF is a safe adjuvant and worthy for further clinical evaluation.

In conclusion, we demonstrate that rLF is an antigen as well as an adjuvant for promoting mucosal and systemic immune responses. rLF is a broad and comprehensive adjuvant that can be formulated with different vaccine types.

## Methods

### Construction of expression vectors

Based on the amino acid sequence of FLIPr (accession number BAB57318), the DNA sequence encoding FLIPr was optimized for *Escherichia coli* codon usage and fully synthesized by Genomics Co. (New Taipei City, Taiwan). The FLIPr gene was cloned into the NdeI and XhoI sites of the expression vector pET- 22b(+) (Novagen, Madison, WI) to produce the plasmid pF. To clone and express LF, the D1 domain and the lipid signal peptide of the lipoprotein Ag473^19^ were cloned into the NdeI and BamHI sites of the pET-22b(+) expression vector (Novagen, Madison, WI, USA) to obtain the plasmid pLipo. The forward primer (5ʹ-ACTGCGGGATCCTTTTTTAGCTA TGAATGG-3ʹ, BamHI site is underlined) and the reverse primer (5ʹ-GTGGTGCTCGAGATCCCAATAAATGCTATC-3ʹ, XhoI site is underlined) were used to amplify the FLIPr gene. The PCR product was cloned into the BamHI and XhoI sites of the pLipo plasmid to produce the plasmid pLF. As a result, the C-termini of rF and rLF contained a hexahistidine tag (His-tag).

### Production and purification of recombinant proteins

The production and purification of rOVA and rZE3 were described previously^27,48^ (see Supplementary Methods for detailed information). To express recombinant FLIPr (rF), the *E. coli* BL21(DE3) strain was transformed with pF. The transformed cells were cultured with LB broth at 23 °C overnight. The overnight culture was scaled up 25 times the original volume in a 2 L shake flask and incubated at 37 °C until the OD_600_ reached 0.4. Then, the culture was cooled to 20 °C, and protein expression was induced (OD_600_ = 0.9) by adding 1 mM IPTG, followed by incubation at 20 °C for 24 h. rF was purified by disrupting the harvested cells in a French press (Constant Systems, Daventry, UK) at 27 Kpsi in homogenization buffer [20 mM Tris (pH 8.0), 40 mM sucrose, 400 mM NaCl and 10% glycerol]. The cell lysate was clarified by centrifugation at 119000 × g for 40 min at 4 °C. Most of the rF was solubilized. The extracted fraction was loaded onto immobilized metal affinity chromatography (IMAC) columns (2.5 cm i.d. × 10.0 cm) (BIO-RAD, Hercules, CA, USA) containing 20 ml of Ni-NTA resin (Qiagen, San Diego, CA) to purify the rF. The column was washed with a 40-fold column volume of the same homogenization buffer and followed by a 50-fold column volume of the same buffer containing 10 mM imidazole. Then, a 100-fold column volume of 10 mM Na_2_HPO_4_ buffer (pH 9.6) containing 0.1% Triton X-114 was washed to remove the lipopolysaccharide. Next, the column was washed with the same buffer without 0.1% Triton X-114 to remove the residual detergent, and the rF was eluted with elution buffer [10 mM Na_2_HPO_4_ (pH 9.6), 10 mM imidazole]. The eluted rF was dialyzed to 10 mM Na_2_HPO_4_ (pH 9.6) three times for at least 6 h each time.

To express the rLF, the *E. coli* C43(DE3) strain was transformed with pLF. The transformed cells were cultured with LB broth at 37 °C overnight. The overnight culture was scaled up by 50 times the original volume in a 2 L shake flask and incubated at 37 °C until the OD_600_ reached 0.6. Protein expression was induced (OD_600_ = 0.6) by adding 1 mM IPTG, followed by incubation at 20 °C for 24 h. rLF was purified by disrupting the harvested cells in a French press (Constant Systems, Daventry, UK) at 27 Kpsi in homogenization buffer [20 mM Tris (pH 8.0), 40 mM sucrose, 400 mM NaCl and 10% glycerol]. The cell lysate was clarified by centrifugation at 119000 × g for 40 min at 4 °C. Most of the rLF was present in inclusion bodies. rLF was then solubilized with extraction buffer [10 mM Na_2_HPO_4_ (pH 9.0) and 1% Triton X-100]. The extracted fraction was loaded onto immobilized metal affinity chromatography (IMAC) columns (2.5 cm i.d. × 10.0 cm) (BIO-RAD, Hercules, CA) containing 20 ml of Ni-NTA resin (Qiagen) to purify rLF. The column was washed with the extraction buffer and the same buffer containing 20 mM imidazole. Then, the rLF was eluted with homogenization buffer containing 500 mM imidazole. The eluted rLF was dialyzed to 20 mM Tris (pH 8.0) three times for at least 6 h each time. After dialysis, the rLF was loaded onto a 20-ml Q Sepharose fast flow column (GE Healthcare, Little Chalfont, Buckinghamshire, UK). The column was washed with dialysis buffer containing 200 mM NaCl and then washed with a 100-fold column volume of dialysis buffer containing 0.1% Triton X-114 to remove the lipopolysaccharide. Next, the column was washed without 0.1% Triton X-114 to remove the residual detergent, and rLF was eluted with elution buffer [10 mM Na_2_HPO_4_ (pH 9.0), 300 mM NaCl and 8 M urea]. The eluted rLF was dialyzed to 10 mM Na_2_HPO_4_ (pH 9.0) three times for at least 6 h each time.

The endotoxin levels of the purified rF and rLF were determined using the limulus amebocyte lysate (LAL) assay (Associates of Cape Cod, Inc., Cape Cod, MA), and the resulting endotoxin levels were <30 EU/mg. After dialysis, the rF and rLF were lyophilized and stored at −20 °C. The fractions from each step were analyzed by SDS-PAGE and immunoblotted with anti-FLIPr (homemade) and anti-His tag antibodies with 1:1000 dilution (Bio-Rad, Cat#MCA1396G, clone AD1.1.10). The N-terminal fragments of rLF were obtained and identified after trypsin digestion of rLF. The identification of the lipid moiety in rLF was performed on a Waters® MALDI micro MX™ mass spectrometer and described previously^21^ (See Supplementary Methods for detailed information).

### Effect of LF on dendritic cell activation

The femurs and tibiae of C57BL/6 mice were obtained, and the bone marrow cells were dispersed by vigorous pipetting. The cells were then treated with lysis buffer to remove red blood cells, and the isolated bone marrow cells were resuspended (5 × 10^5^ cells/mL) in RPMI-1640 supplemented with 10% (v/v) heat-inactivated fetal bovine serum, penicillin/streptomycin (50 units/mL), l-glutamine (2 mM), HEPES (20 mM), and β-mercaptoethanol (50 µM) at 37 °C under 5% CO_2_. On days 0 and 3, granulocyte macrophage colony stimulating factor (200 units/mL) was added to the cultures. Cultured cells were harvested on day 6. One-mL aliquots of suspended BMDCs (1 × 10^6^ cells/mL) were seeded into 24-well plates and stimulated with rF or rLF (5 µg/mL). The supernatants were collected at 20 h after stimulation. The TNF-α (Invitrogen, Cat#88-7324-88), IL-6 (Invitrogen, Cat#88-7064-88), IL-12p70 (Invitrogen, Cat#88-7121-88), IL-23p19 (Invitrogen, Cat#88-7230-88), IL-1α (Invitrogen, Cat#88-5019-88), and IL-1β (Invitrogen, Cat#88-7013-88) levels in the supernatants were determined using specific cytokine kits purchased from Invitrogen (Waltham, MA). Cells were harvested for surface marker staining with APC/Cyanine7 anti-CD11c (BioLegend, Cat#117323, clone N418), FITC MHCII (BioLegend, Cat#107606, clone M5/114.15.2), PE anti-CD40 (BioLegend, Cat#124610, clone 3/23), PerCP/Cyanine5.5 anti-CD80 (BioLegend, Cat#104722, clone 16-10A1) and PE/Cyanine7 anti-CD86 (BioLegend, Cat#105014, clone GL-1) monoclonal antibodies after 20 h of stimulation. Staining antibodies were all used at a 1:100 dilution. The expression of surface markers was analyzed by flow cytometry (Attune^TM^ NxT, Thermo Fisher Scientific) on gated CD11c^+^ MHCII^+^ cell populations. The data were acquired using Attune^TM^ NxT software and analyzed using FlowJo 10.8.1 software.

### Isolation of human neutrophils

Venous blood was collected from healthy volunteers into tubes containing preservative-free heparin (20 U/ml final). The venous blood was mixed with an equal volume of 3% dextran T-500 in 0.9% NaCl and incubated in an upright position for 30 min at room temperature. The leukocyte-rich plasma (upper layer) was aspirated and saved using a pipette and syringe. Then, the leukocyte-rich plasma was placed on top of Ficoll-Hypaque solution at a 2:1 plasma/Ficoll-Hypaque solution ratio using a pipette or a syringe fitted with a long blunt-end needle, and centrifugation was then performed for 40 min at 395 × g at 20 °C with no brake. After centrifugation, the top layer and the Ficoll-Hypaque layer were aspirated, leaving the neutrophil/RBC pellet. Each pellet was then resuspended in 20 ml of cold 0.2% NaCl to lyse the RBCs and restore isotonicity by adding 20 ml of ice-cold 1.6% NaCl. The cells were centrifuged for 6 min at 200 × g at 4 °C and resuspended in ice-cold PBS. The isolated human neutrophils were determined by staining with PE-conjugated anti-CD16 antibody (BioLegend, Cat#980102, clone 3G8) and PE-Cy7-conjugated anti-CD66b antibody (BioLegend, Cat#305116, clone G10F5), and the purity was determined by flow cytometry. Staining antibodies were all used at a 1:100 dilution. Human neutrophils with a purity greater than 90% were required. All study procedures were approved by the Research Ethics Committee of National Health Research Institutes (Institutional Review Board numbers: 2107290066).

### Phagocytosis

Phagocytosis was measured with FITC-labeled *Staphylococcus aureus* strain ATCC 25923. The bacteria were mixed with 10% complement-inactivated (30 min at 56 °C) serum from PBS-, rF-, or rLF-immunized mice (pooled from 9 mice) for opsonization. The bacteria-serum complex was then mixed with or without 30 µg/mL rF (as an inhibitor to inhibit phagocytosis) for 20 min at 37 °C. Human neutrophils (determined as CD16^+^ CD66b^+^ cells and as described in the preceding paragraph) were then added at a 10:1 bacteria/cell ratio and incubated for the indicated time at 37 °C with a 750 rpm shaker. The reaction was stopped with ice-cold paraformaldehyde (3.7%). After 2 washes with PBS, cell-associated fluorescent bacteria were analyzed by flow cytometry. Phagocytosis was determined as the relative geometric mean fluorescence intensity of cells with fluorescent bacteria. Each relative value is compared to the geometric mean fluorescence intensity at an incubation time of 2.5 min of the groups without rF treatment.

### Mice

C57BL/6 and BALB/c mice were obtained from the National Laboratory Animal Breeding and Research Center (Taipei, Taiwan). AG129 mice, immunocompromised mice lacking the receptor for types I and II IFN (IFN α/β/γ), were bred at the Laboratory Animal Center of the National Health Research Institutes (NHRI). All the mice were housed at the Laboratory Animal Center of the NHRI. All the animal studies were approved and were performed in compliance with the guidelines of the Animal Committee of the NHRI. Mice were euthanized by controlled administration of carbon dioxide inhalation. Blood samples for serum collection were obtained using the submandibular vein method. All intranasal administrations, blood sample collection, and vaginal lavage were performed under anaesthetization using isoflurane inhalation.

### Analysis of antigen uptake by NALT dendritic cells

rOVA was labeled with an Alexa Fluor 700 labeling kit (Abcam, Cambridge, UK). Groups of C57BL/6 mice (6–8 weeks of age) were intranasally administered 30 µg of Alexa Fluor 700-labeled rOVA or Alexa Fluor 700-labeled rOVA adjuvanted with 10 μg of rF or rLF. At 18 h after administration, a single NALT cell suspension (pooled from 3 mice) was made as described previously^49^ (See Supplementary Methods for detailed information). The Zombie Violet™ Fixable viability kit (Biolegend, San Diego, CA) was used to evaluate the viability of NALT cells by flow cytometry. Lymphocytes were distinguished by staining with PE /Cyanine7-conjugated anti-CD45 antibody (BioLegend, Cat#103114, clone 30-F11). Dendritic cells were distinguished by staining with Alexa Fluor® 488-conjugated anti-MHCII antibody (BioLegend, Cat#107616, clone M5/114.15.2) and PE-conjugated anti-CD11c antibody (BioLegend, Cat#117307, clone N418). Staining antibodies were all used at a 1:100 dilution. The frequency of the antigen-positive (Alexa Fluor 700 + ) DCs in draining NALT was analyzed by flow cytometry.

### Immunization and sample collection

Groups of mice (6–8 weeks of age) were vaccinated three times with each vaccine candidate at a two-week interval by intranasal administration. The dosage of each vaccine candidate was 30 µg of each antigen (rOVA and rZE3) or 5.6 × 10^4^ FFU iH7N9 (containing 0.475 µg HA). The above antigens were mixed with 10 µg of rLF in some groups. PBS was used as a control. Serum and vaginal lavage samples were collected from each mouse at different time points as indicated. BALF and NW samples were collected 6 weeks after the first vaccination.

### Measurement of antibody titers

The antigen-specific IgG and IgA titers in the indicated samples were determined by titration as previously described^49^ (See Supplementary Methods for detailed information).

### Enzyme-linked immunospot (ELISPOT) assays

To detect and quantify individual OVA-specific antibody-secreting cells in bone marrow, bone marrow cells were analyzed using ELISPOT. The mice were sacrificed 20 weeks after the first immunization, and bone marrow cells were prepared. In brief, 96-well plates with PVDF membranes (Millipore, Burlington, MA) were coated with rOVA and incubated at 4 °C for 18 h. The plates were washed twice and blocked with RPMI medium supplemented with fetal bovine serum (10%) for 1 h to prevent nonspecific binding. Bone marrow cells were seeded at a concentration of 1 × 10^5^ cells/well for 3 days. After incubation, the cells were removed from the plates by washing 3 times with 0.05% (w/v) Tween 20 in PBS (PBST). Biotinylated anti-mouse IgG antibody with 1:300 dilution (Sigma-Aldrich, St. Louis, MO. Cat#B8520) was added to each well. The plates were then incubated at 37 °C for 2 h. Then, the plates were washed with PBST, and avidin-horseradish peroxidase complex reagent was added for 45 min of incubation at room temperature. After washing with PBST, 3-amine-9-ethyl carbazole (Sigma-Aldrich, St. Louis, MO) staining solution was added to each well to develop the spots. The reaction was stopped by washing the plates with water. The spots were counted using an ELISPOT reader (Cellular Technology Ltd.).

To detect the number of IFN-γ-producing cells in the spleen, mouse IFN-γ ELISPOT kits (BD Biosciences, San Jose, CA. Cat#551083) were used. The mice were sacrificed 6 or 20 weeks after the first immunization, and splenocytes were prepared. The procedures were performed according to the manufacturer’s instructions. The splenocytes (5 × 10^5^ cells/well) were seeded into 96-well plates and incubated with 10 µg/mL OT-1 (OVA_257−264_, SIINFEKL), OT-2 (OVA_323−339_, ISQAVHAAHAEINEAGR) peptides, OT-1 control peptides (QYEGDGSPCKIPFEI, derived from dengue virus), OT-2 control peptides (GRLITVNPIVTEKDS, derived from dengue virus), or media (no stimulation) for three days. The spots were determined as previously described^48^ (See Supplementary Methods for detailed information).

### Quantification of cytokine release

The spleens were removed to create single-cell suspensions at 6 weeks after the first immunization. Splenocytes were added to each well of a 24-well plate and were further stimulated with rOVA or BSA at 10 µg/mL. One well was set up for each stimulation. Following culturing for 3 days, cell-free supernatants were harvested and stored at −80 °C. The IFN-γ (Invitrogen, cat# 88-7314-88), IL-13 (Invitrogen, cat# 88-7137-88) and IL-17A (Invitrogen, cat# 88-7371-88) levels were measured using ELISA kits according to the manufacturer’s instructions.

### Animal challenge

The challenge to vaccine-immunized AG129 mice was via the intraperitoneal injection route^49^. The dosage of the Zika virus (PRVABC59) challenge was 80 FFUs in 0.2 mL of PBS per AG129 mouse.

### Focus-forming assays and focus reduction neutralization tests (FRNT)

Plasma from challenged mice at the indicated time points was diluted using 10-fold serial dilutions (starting at 1:10). Viral titers in the plasma were determined by focus-forming assays as previously described^27^ (See Supplementary Methods for detailed information). If the viral titers were less than 2.3 log10 FFU/mL (equivalent to the detection limit here), a value of 1.0 was assigned for calculation purposes. Neutralizing antibody titers were determined by FRNT as previously described^27^ (See Supplementary Methods for detailed information). If the viral titers were less than 3.0 log2 (equivalent to the detection limit here), a value of 2.0 was assigned for calculation purposes.

### Production and inactivation of H7N9

The H7N9 (A/Guangdong/17SF003/2016) influenza virus was produced from adherent MDCK cells and inactivated with formaldehyde (final 0.01%) at 37 °C for 24 h^50^. The production and manufacturing of the inactive H7N9 are carried out by the Bioproduction Plants of the National Institute of Infectious Diseases and Vaccinology at the National Health Research Institutes in Taiwan.

### Hemagglutination inhibition assays

To remove nonspecific inhibitors of HA, reference anti-serum and complement-inactive serum from vaccine-immunized mice were diluted 1:4 with 0.85% receptor-destroying enzyme (RDE) (Sigma-Aldrich), followed by an 18 h incubation at 37 °C. The RDE-treated serum was then diluted 1:2.5 with phosphate-buffered saline. The nonspecific agglutinins in the RDE-treated sera were removed by hemadsorption against turkey RBCs (with a final concentration of 4.5%) for 1 h at 4 °C. To check the hemoadsorption efficiency, adsorbed serum was mixed with an equal volume of 0.5% RBCs. Before the HI assay, the virus was diluted with PBS to make a working solution containing 8 HAU/50 µl, and back titration was performed to verify the accuracy of the HA units (=8 HAU/50 µl). When ready, the 25 µl per well adsorbed sera in V-well microtiter plates were serially diluted 2-fold (starting from 1:10) and incubated with 25 µl of 4 HA units of virus per well for 15 min at room temperature. Then, 50 μl of 0.5% turkey RBCs was added, and the reaction mixture was incubated for 40 min at RT until the cells in the RBC control wells had fully settled. The inhibition of HA was examined by visualizing the RBC buttons or teardrop formation upon plate tilting. HI titers were defined as the reciprocal highest dilution of serum that completely prevented HA.

### Data analysis

Values are expressed as the means ± SEM. The Kruskal-Wallis test with Dunn’s multiple comparison was used to compare differences for more than two groups. Statistical analysis was performed using GraphPad Prism software version 6.01 (GraphPad Software, San Diego, CA). Differences with *p* < 0.05 were considered statistically significant.

### Reporting summary

Further information on research design is available in the Nature Research Reporting Summary linked to this article.

## Supplementary information

Supplementary Information

REPORTING SUMMARY

## Data Availability

All relevant data are presented in this paper.
