# Supplementary material for: Recombinant lipidated FLIPr effectively enhances mucosal and systemic immune responses for various vaccine types
Source: NPJ Vaccines. 2023 Jun 2;8:82. doi: 10.1038/s41541-023-00680-4 (PMC10236402; doi:10.1038/s41541-023-00680-4)
Supplement: Supplementary file 1 — Supplementary Information [file 41541_2023_680_MOESM1_ESM.pdf]

Supplementary Materials for

Recombinant lipidated FLIPr is a potent adjuvant that enhances mucosal and systemic immune responses for various vaccine types

Ming-Shu Hsieh[1], Mei-Yu Chen[1], Chia-Wei Hsu[1], Yu-Wen Tsai[1], Fang-Feng Chiu[1], Cheng-Lung Hsu[2], Chang-Ling Lin[1], Chiao-Chieh Wu[1], Ling-Ling Tu[1], Chen-Yi Chiang[1], Shih-Jen Liu[1,3,4], Ching-Len Liao[1], Hsin-Wei Chen[1,3,4*]

[1]National Institute of Infectious Diseases and Vaccinology, National Health Research Institutes, Miaoli, Taiwan.
[2]Division of Hematology-Oncology, Department of Internal Medicine, Chang Gung Memorial Hospital, Chang Gung University, Taoyuan, Taiwan.
[3]Graduate Institute of Biomedical Sciences, China Medical University, Taichung, Taiwan.
[4]Graduate Institute of Medicine, Kaohsiung Medical University, Kaohsiung, Taiwan.

*Correspondence:
Hsin-Wei Chen (Email: chenhw@nhri.org.tw)

Included:
Supplementary Figure 1-3
Supplementary Table 1
Supplementary Methods

Supplementary Figure 1

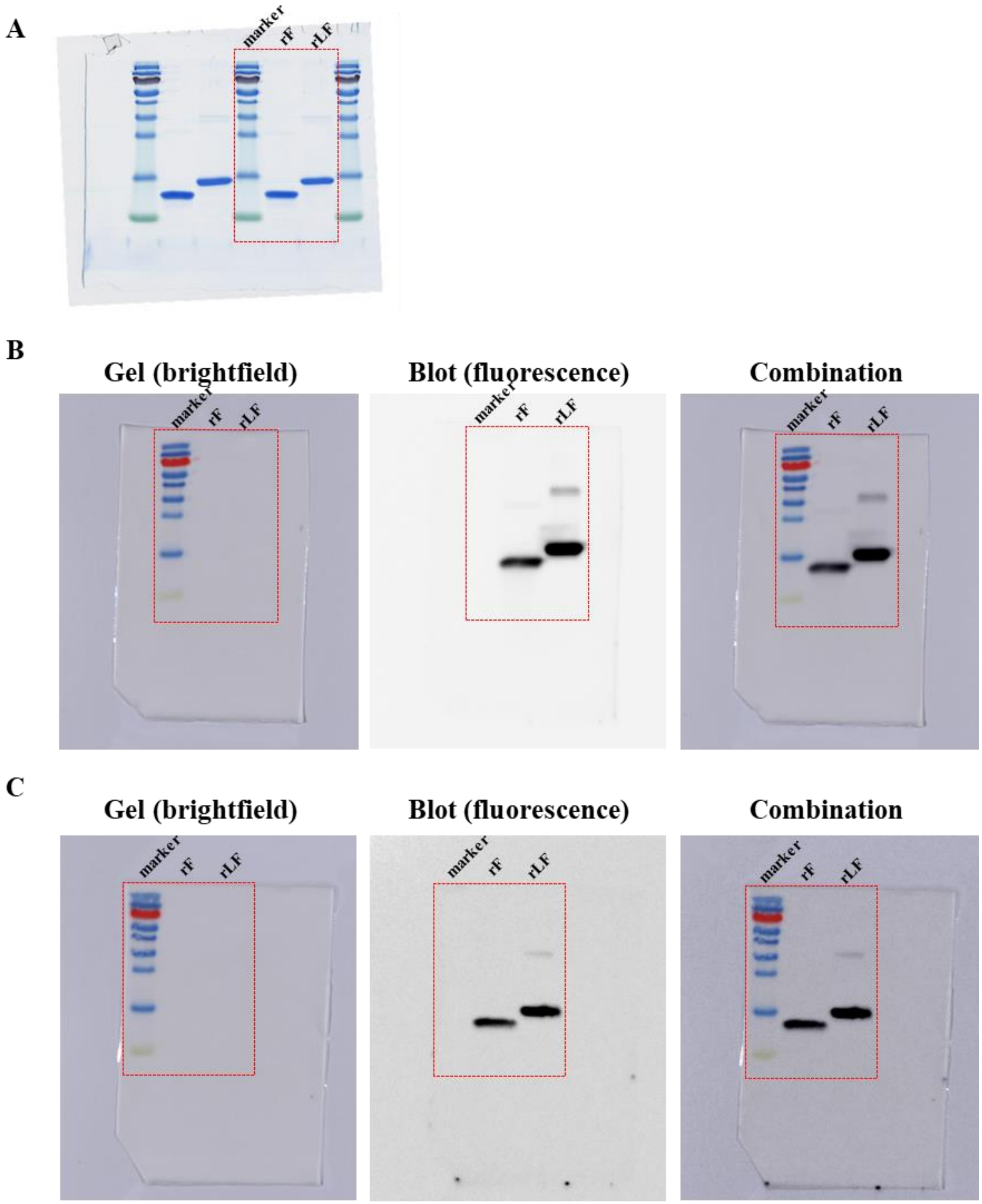

Supplementary Figure 1. Raw images for edited Figure 1A. (A) the left panel. (B) the middle panel. (C) the right panel.

Supplementary Figure 2

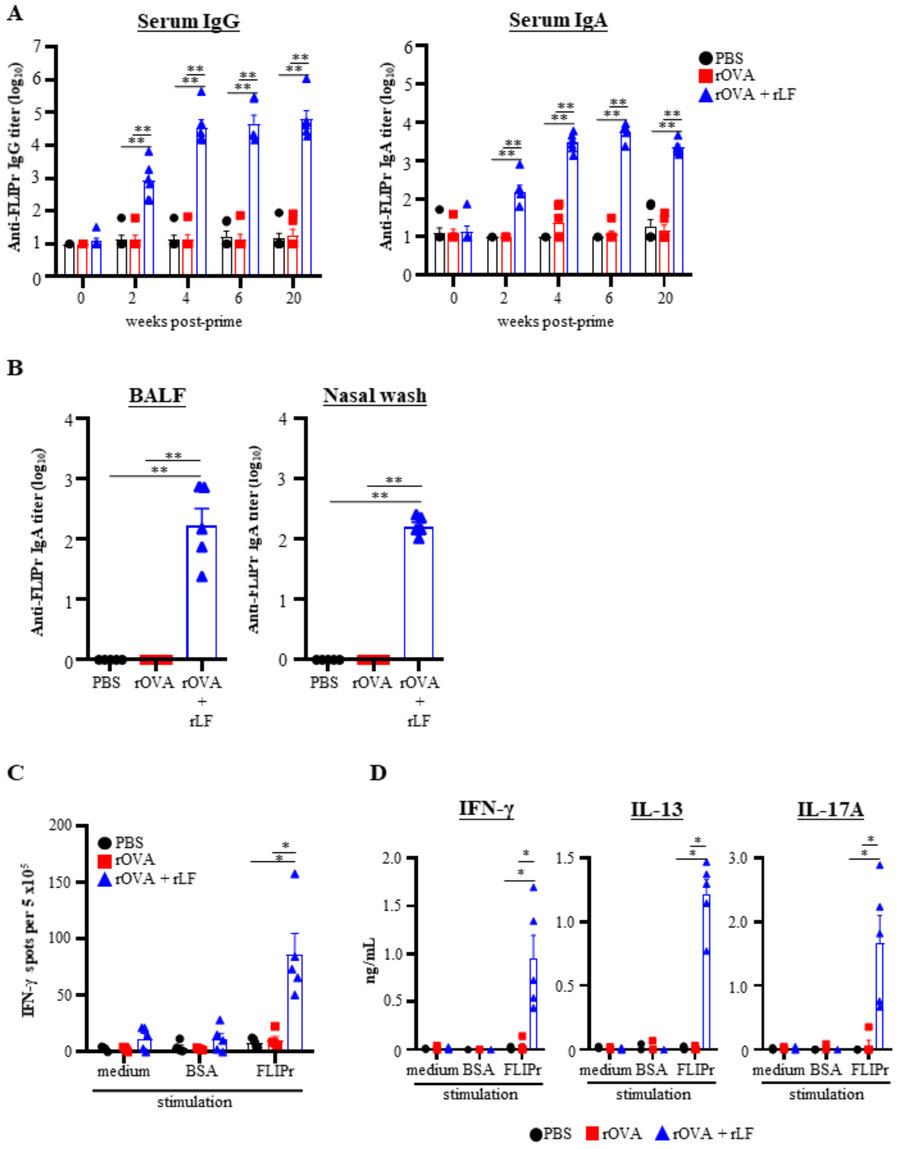

Supplementary Figure 2. FLIPr-specific antibody and T cell responses induced by intranasal administration of rOVA plus rLF. Groups of C57BL/6 mice (n=5 or 6/group) were intranasally immunized three times with 30 µg of rOVA or rOVA mixed with 10 µg of rF at 2-week intervals. Mice immunized with PBS alone served as controls. (A) Sera were collected at the indicated time points. FLIPr-specific IgG and IgA titers were assessed by ELISA. (B) Samples of BALF and nasal wash were collected from mice 6 weeks after the first vaccination. Reactivity of FLIPr-specific IgA antibody titers in BALF and nasal wash was assessed by ELISA. (C) Splenocytes were harvested 6 weeks after the first vaccination. Cells were cultured and stimulated with FLIPr, BSA, or medium alone for 3 days in an anti-INF-γ-coated 96-well

ELISPOT plate. IFN-γ responses were measured using ELISPOT assay and are expressed as spot-forming units per 5×10^5 cells. (D) Splenocytes were cultured and stimulated with rFLIPr for 3 days. Stimulation with BSA or medium alone served as controls. The supernatants were collected to evaluate levels of IFN-γ, IL-13 and IL-17A by ELISA. Data represent the mean ± SE of the mean. The results shown are from one of two representative experiments. Statistical significance was determined using the Kruskal-Wallis test with Dunn's multiple comparison test. $*p < 0.05$; $**p < 0.01$.

Supplementary Figure 3

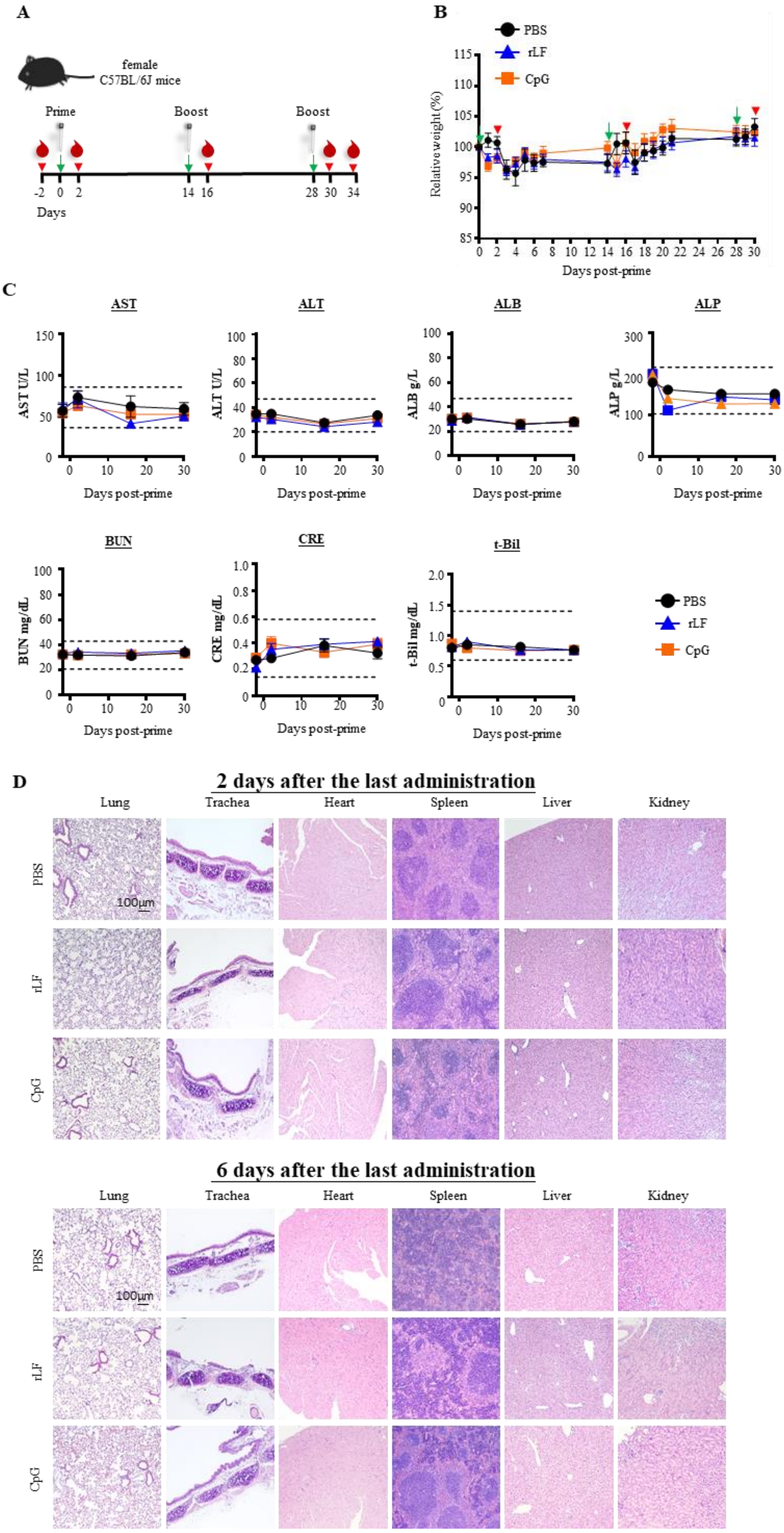

Supplementary Figure 3. Safety assessment of rLF. The experimental flow chart is shown (A). Groups of 6-weeks age C57BL/6 mice (n=6/group) were intranasally administrated three times with 10 μg rLF or CpG at 2-week intervals (on day 0, 14, and 28). Mice administrated with PBS alone served as controls. The blood samples were collected at 2 days before prime (day -2) and 2 days after each immunization (day 2, 16, and 30). Mice were sacrificed and whole blood samples collected on the 2 (n=3) or 6 (n=3) days after the last administration. The time point of administration is marked by the green arrow, and the time point of blood collection is marked by the red arrow head. (B) Body weights were measured. Relative weight values are based on weight at day 0 as 100% for comparison. (C) Serum biochemical analytes were measured after administration. All serologic parameters from individual mice were determined by transfer serum to FUJI DRI-CHEM slide, followed by analysis using a FUJI DRI CHEM NX500i Chemistry System. Dashed lines on the y-axis indicated the normal comparable range of previously reported values (Hum Gene Ther. 2002, 13(1):155-61; Comparative Medicine. 2004, 54(2):176-8; PLoS One. 2008, 3(11):e3772.). AST = aspartate transaminase, ALT =alanine transaminase, ALB = albumin, ALP = Alkaline Phosphatase, BUN = Blood Urea Nitrogen, CRE = Creatinine; t-Bil = Total Bilirubin. (D) Histopathologic analysis after administration. Mice were sacrificed at 2 (n=3) (up panel) and 6 (n = 3) (lower panel) days after the last administration. The lungs, trachea, heart, spleen, liver, and kidney were subjected to histologic analysis by H and E staining.

Supplementary Table 1

Supplementary Table 1. Hematological parameters after administration with rLF.

Parameters (unit)	Group (mean ± SD) n=3 Day 30 PBS	rLipo- FLIPr	CpG	Day 34 PBS	rLipo- FLIPr	CpG	Reference range
WBC(10^3/ mm^3)	9.49 ± 4.91	7.56 ± 2.82	7.86 ± 2.79	7.84 ± 0.48	9.15 ± 0.98	6.72 ± 0.36	2.20–11.53
RBC (10^6/mm^3)	7.33 ± 0.55	6.87 ± 0.53	7.39 ± 1.34	7.68 ± 0.24	7.12 ± 0.19	7.49 ± 0.55	3.47–11.73
HGB (g/dL)	11.7 ± 0.68	10.63 ± 1.04	11.53 ± 2.19	12.4 ± 0.45	11.7 ± 0.43	12.33 ± 0.54	5.70–17.00
HCT (%)	36.77 ± 2.46	34.57 ± 2.78	36.8 ± 6.84	38.67 ± 1.6	37.13 ± 0.21	37.6 ± 2.05	16.20–58.30
PLT (10^4/mL)	108.73 ± 11.41	110.9 ± 17.61	115.6 ± 14.24	130.76 ± 15.8	140.63 ± 52.63	173.53 ± 7.56	99.00-184.00

The blood samples were collected at 2 or 6 days after last administration (day 30 and 34). Hematological assays were conducted with Mindray BC-5000 Vet.
Reference range indicates the normal range values for female C57BL/6 mice in previous reports. (Hum Gene Ther. 2002, 13(1):155-61; PLoS One. 2008, 3(11):e3772.).

Supplementary Methods

Production and purification of rOVA. The expression plasmid pOVA was used to transform *E. coli* strain BL21 (DE3) (Invitrogen, Carlsbad, CA, USA) for rOVA expression. After culturing the transformed cells in LB broth overnight at 20°C, they were scaled up to 37°C until the optical density reached 0.6 - 1.0. To induce rOVA expression, 1 mM isopropylthiogalactoside (IPTG) was added, and the cells were further incubated at 12°C for 3 days. Cells were collected and disrupted using a French press (Constant Systems, Daventry, UK) at 27 Kpsi in a homogenization buffer containing 20 mM Tris (pH 8.0), 50 mM sucrose, 500 mM NaCl and 10% glycerol. After centrifugation (80,000×g for 40 min), the supernatant was loaded onto a column (BIO-RAD, Hercules, CA, USA, 2.5 cm i.d. × 10.0 cm) containing 20 ml Ni-NTA resin (Qiagen, San Diego, CA, USA) and washed with the extraction buffer and then the same buffer containing 20 mM imidazole. Then, rOVA was eluted with the homogenization buffer containing 300 mM imidazole. The eluted rOVA was dialyzed to 20 mM Tris (pH 8.0) three times for at least 6 h each time. After dialysis, the rOVA was loaded onto a 20-ml Q Sepharose fast flow column (GE Healthcare, Little Chalfont, Buckinghamshire, UK) and washed with dialysis buffer. The rOVA was eluted with dialysis buffer containing 150 mM NaCl. Mustang E membrane (Pall corporation, NY, USA) was used to remove lipopolysaccharide (LPS) and to exchange the buffer with phosphate buffer saline (PBS). The amount of residual LPS in the rOVA preparations was tested for endotoxin levels using a Limulus amebocyte lysate (LAL) assay (Associates of Cape Cod, Inc., Cape Cod, MA), which showed a residual endotoxin concentration of less than 10 EU/mg. Following endotoxin removal, rOVA was dialyzed against 0.01 M dibasic sodium phosphate, lyophilized, and stored at -20°C.

Production and purification of rZE3. For expression of rZE3, plasmid encoding Zika virus envelope protein domain III (pZE3) was transformed into *E. coli* BL21 (Invitrogen, Carlsbad, CA). After transformation, the *E. coli* were cultured at 37°C overnight. To scale up protein production, 20 ml of the overnight culture was transferred to a 2-L shaker flask containing 1 L of medium, and then incubated at 37°C for 4 hours. Once the OD600 of the culture reached 0.6, protein expression was induced by adding 1 mM IPTG and incubating for 20 hours at 20°C. Cells were collected and disrupted using a French press (Constant Systems, Daventry, UK) at 27 Kpsi in a homogenization buffer containing 20 mM Tris (pH 8.0), 50 mM sucrose, 500 mM NaCl and 10% glycerol. After centrifugation (80,000×g for 40 min), the cell lysate was clarified. It was observed that most of the rZE3 was present in the

inclusion bodies. The extraction of rZE3 was carried out using an extraction buffer (0.02 M Tris, 0.05 M sucrose, 0.5 M NaCl, 10% glycerol, and 3 M GuHCl). To purify rZE3, the solubilized fraction was loaded onto immobilized metal affinity chromatography (IMAC) columns (QIAgen, Hilden, Germany). Subsequently, the eluate obtained from the IMAC column was further purified using an anion exchange column (Ni-NTA superflow slurry). The refined fraction was subjected to endotoxin removal by passing it through an E membrane (Pall Co., USA). The purified rZE3 fraction was tested for endotoxin levels using a Limulus amebocyte lysate (LAL) assay (Associates of Cape Cod, Inc., Cape Cod, MA), which showed a residual endotoxin concentration of less than 10 EU/mg. Following endotoxin removal, rZE3 was dialyzed against 0.01 M dibasic sodium phosphate, lyophilized, and stored at -20°C.

Identification of the lipid moiety in rLF. rLF was treated with trypsin (Sigma, St. Louis, MO), followed by purification of the resulting mixture using a ZipTip (Millipore, Massachusetts). A 1 μL sample of the tryptic fragments that had been polished using the ZipTip was combined with 1 mL of a saturated solution of α-cyano-4-hydroxycinnamic acid in acetonitrile/0.1% trifluoroacetic acid (1:3 vol:vol). The mixture (1 μL) was analyzed using a MALDI micro MX mass spectrometer (Waters, Manchester, UK).

NALT single-cell preparation. The NALT tissue was mechanically disrupted and transferred to conical tubes. Subsequently, tissue pieces were resuspended in 1 mL of RPMI supplemented with 0.4 mg/mL collagenase (Sigma-Aldrich, St. Louis, MO) and incubated at 37°C for 30 minutes. The tissue was then ground, and the resulting suspension was collected and filtered through a 70 μm cell strainer. The cells were collected by centrifugation at 300×g for 5 minutes.

Measurement of antibody titers. Serum samples were serially diluted three-fold (starting at the indicated dilution) and added to 96-well plates coated with the antigen. 1:5000 diluted peroxidase-conjugated anti-mouse IgG Fc IgG fraction antibody(MP Biomedicals, Cat#0855554) and 1:1500 diluted HRP Goat anti-Mouse IgA Cross-Adsorbed Secondary Antibody (Invitrogen, cat# 62-6720) were used to detect bound IgG and IgA, respectively. After washing with PBS, a substrate 3,3',5,5'-tetramethylbenzidine was added, and the absorbance at 450 nm was measured using an ELISA reader. The endpoint titer was determined as twice the mean of the background OD value. Titers were calculated by interpolation from the titration curve, unless the OD value was less than twice the mean of the background at the starting

dilution.

Enzyme-linked immunospot (ELISPOT) assays. After incubation, the splenocytyes were discarded from the plates by washing three times with 0.05% (w/v) Tween 20 in PBS. The wells were first treated with biotinylated detection antibody (0.1 ml/well) and incubated at 37°C for 2 hours. The plates were then washed following the same steps as before and the avidin-horseradish peroxidase complex reagent was added. After incubating the plates at room temperature for 45 minutes, the wells were washed three times with 0.05% (w/v) Tween 20 in PBS followed by three washes with PBS alone. To develop the spots, staining solution (3-amine-9-ethylcarbazole, Sigma-Aldrich) was added to the wells (0.1 ml/well), and the plates were incubated for 1 hour. The reaction was stopped by placing the plates under tap water, and the spots were determined using an ELISPOT reader (Cellular Technology Ltd., Shaker Heights, OH, USA).

Focus-forming assays. Plasma samples from the challenged mice were diluted in 10-fold serial dilutions, with a starting dilution of 1:10. The diluted plasma samples were used to infect a monolayer of Vero cells in 24-well plates at 37°C. After incubating for 3 hours, a medium overlay containing 2.5% fetal bovine serum and 0.8% methylcellulose in DMEM was added. The infected monolayer was incubated at 37°C for 55 hours, after which the overlay medium was removed. The cells were then fixed in 3.7% formaldehyde/PBS for 15 minutes, permeabilized with 0.1% nonidet P40/PBS for 15 minutes, and blocked with 3% bovine serum albumin/PBS for 15 minutes. The 1:8000 diluted HB122 anti-ZV antibody (produced and purify from hybridoma D1-4G2-4-1, ATCC HB-112) was used to identify ZV-infected Vero cells. After washing with PBS, a 1:10000 diluted horseradish peroxidase-conjugated goat anti-mouse IgG (H+L) antibody (ThermoFisher, Cat#31430) was used to identify antibody-bound cells. The infected cells were visualized using TMB.

Focus reduction neutralization tests (FRNT). The serum samples were heat-inactivated and diluted 2-fold in serial dilutions (starting at 1:8). Zika virus was then incubated with the serum samples at 4°C overnight in a final volume of 200 μL. The mixture was added to monolayers of Vero cells in 24-well plates, and the focus-forming units (FFUs) were determined using focus-forming assays. The $FRNT_{50}$ neutralizing antibody titer was determined as the highest dilution resulting in a 50% reduction in FFUs compared to the FFUs of negative control samples containing virus alone.
