# Supplementary material for: Recombinant lipidated FLIPr effectively enhances mucosal and systemic immune responses for various vaccine types
Source: NPJ Vaccines. 2023 Jun 2;8:82. doi: 10.1038/s41541-023-00680-4 (PMC10236402; doi:10.1038/s41541-023-00680-4)
Supplement: Supplementary file 2 — REPORTING SUMMARY [file 41541_2023_680_MOESM2_ESM.pdf]

nature portfolio

Corresponding author(s): Hsin-Wei Chen

Last updated by author(s): Jan 31, 2023

Reporting Summary

Nature Portfolio wishes to improve the reproducibility of the work that we publish. This form provides structure for consistency and transparency in reporting. For further information on Nature Portfolio policies, see our Editorial Policies and the Editorial Policy Checklist.

Statistics

For all statistical analyses, confirm that the following items are present in the figure legend, table legend, main text, or Methods section.

n/a	Confirmed	
☐	☒	The exact sample size (*n*) for each experimental group/condition, given as a discrete number and unit of measurement
☐	☒	A statement on whether measurements were taken from distinct samples or whether the same sample was measured repeatedly
☐	☒	The statistical test(s) used AND whether they are one- or two-sided<br>*Only common tests should be described solely by name; describe more complex techniques in the Methods section.*
☒	☐	A description of all covariates tested
☒	☐	A description of any assumptions or corrections, such as tests of normality and adjustment for multiple comparisons
☐	☒	A full description of the statistical parameters including central tendency (e.g. means) or other basic estimates (e.g. regression coefficient) AND variation (e.g. standard deviation) or associated estimates of uncertainty (e.g. confidence intervals)
☐	☒	For null hypothesis testing, the test statistic (e.g. *F*, *t*, *r*) with confidence intervals, effect sizes, degrees of freedom and *P* value noted<br>*Give P values as exact values whenever suitable.*
☒	☐	For Bayesian analysis, information on the choice of priors and Markov chain Monte Carlo settings
☒	☐	For hierarchical and complex designs, identification of the appropriate level for tests and full reporting of outcomes
☒	☐	Estimates of effect sizes (e.g. Cohen's *d*, Pearson's *r*), indicating how they were calculated

Our web collection on statistics for biologists contains articles on many of the points above.

Software and code

Policy information about availability of computer code

Data collection	We did not use custom algorithms or software to collect data in this manuscript.
Data analysis	We used GraphPad Prism v.6. For flow cytometry analysis we used FlowJo v10.6.0.

For manuscripts utilizing custom algorithms or software that are central to the research but not yet described in published literature, software must be made available to editors and reviewers. We strongly encourage code deposition in a community repository (e.g. GitHub). See the Nature Portfolio guidelines for submitting code & software for further information.

Data

Policy information about availability of data

All manuscripts must include a data availability statement. This statement should provide the following information, where applicable:

- Accession codes, unique identifiers, or web links for publicly available datasets
- A description of any restrictions on data availability
- For clinical datasets or third party data, please ensure that the statement adheres to our policy

The data that support the findings of this study are available from the corresponding author, [HWC], upon reasonable request.

Human research participants

Policy information about studies involving human research participants and Sex and Gender in Research.

Reporting on sex and gender	To obtain neutrophils from healthy individuals, this study recruited a total 2 male and 1 female volunteers.
Population characteristics	All subject recruited were healthy adults, aged 35 to 45 years old.
Recruitment	Based on opportunity for existing pre-established cohorts, healthy volunteers were recruited from Division of Hematology-Oncology, Department of Internal Medicine, Chang Gung Memorial Hospital ,Chang Gung University, Taoyuan, Taiwan .
Ethics oversight	All study procedures were approved by the Research Ethics Committee of National Health Research Institutes (Institutional Review Board numbers: 2107290066)

Note that full information on the approval of the study protocol must also be provided in the manuscript.

Field-specific reporting

Please select the one below that is the best fit for your research. If you are not sure, read the appropriate sections before making your selection.

☒ Life sciences ☐ Behavioural & social sciences ☐ Ecological, evolutionary & environmental sciences

For a reference copy of the document with all sections, see nature.com/documents/nr-reporting-summary-flat.pdf

Life sciences study design

All studies must disclose on these points even when the disclosure is negative.

Sample size	We used the prior information from our previous studies to determine the sample sizes. Basically, each group had 5-6 animals to achieve statistical power.
Data exclusions	No data were excluded for our analyses.
Replication	Replicate experiments were successful.
Randomization	All animals/samples are simultaneously randomized to the treatment groups without considering any other variable.
Blinding	Most of the data collection are not blinding due to the manpower limitation. The serum biochemical and hematological analysis are blinding.

Reporting for specific materials, systems and methods

We require information from authors about some types of materials, experimental systems and methods used in many studies. Here, indicate whether each material, system or method listed is relevant to your study. If you are not sure if a list item applies to your research, read the appropriate section before selecting a response.

Materials & experimental systems

n/a	Involved in the study	
☐	☒	Antibodies
☐	☒	Eukaryotic cell lines
☒	☐	Palaeontology and archaeology
☐	☒	Animals and other organisms
☒	☐	Clinical data
☒	☐	Dual use research of concern

Methods

n/a	Involved in the study	
☒	☐	ChIP-seq
☐	☒	Flow cytometry
☒	☐	MRI-based neuroimaging

Antibodies

Antibodies used	Immunoblot<br>Mouse anti Histidine tag antibody (Bio-Rad, cat#MCA1396G, clone AD1.1.10), Mouse IgG (H+L) Secondary Antibody (ThermoFisher, cat#31430), Mouse monoclone anti-FLIPr antibody was produced and purify in our lab.<br><br>ELISA

TNF-aplpha mouse elisa kit (Invitrogen, cat# 88-7324-88), IL-6 mouse elisa kit (Invitrogen, cat# 88-7064-88), IL-12p70 mouse elisa kit (Invitrogen, cat# 88-7121-88), IL-23 mouse elisa kit (Invitrogen, cat# 88-7230-88), IL-1 alpha mouse elisa kit (Invitrogen, cat# 88-5019-88), IL-1 beta mouse elisa kit (Invitrogen, cat# 88-7013-88), IFN gamma Mouse Uncoated ELISA Kit (Invitrogen, cat# 88-7314-88), IL-13 Mouse Uncoated ELISA Kit (Invitrogen, cat# 88-7137-88),IL-17A (homodimer) Mouse Uncoated ELISA Kit (Invitrogen, cat# 88-7371-88), peroxidase-conjugated anti-mouse IgG Fc IgG fraction (MP Biomedicals, cat#0855554), HRP Goat anti-Mouse IgA Cross-Adsorbed Secondary Antibody (Invitrogen, cat# 62-6720).

ELISPOT
mouse IFN-γ Elispot set (BD Biosciences, cat# 551083), Anti-Mouse IgG (whole molecule)-Biotin ((Sigma-Aldrich, cat#B8520)

Flow cytometry
FITC anti-mouse I-A/I-E antibody (Biolegend, Cat#107606, clone M5/114.15.2), Alexa Fluor® 488 anti-mouse I-A/I-E antibody (Biolegend, Cat#107616, clone M5/114.15.2),PE anti-mouse CD11c antibody (Biolegend, Cat#117307, clone N418), APC/Cyanine7 anti-mouse CD11c antibody (Biolegend, Cat#117323, clone N418),PE/Cyanine7 anti-mouse CD45 antibody (Biolegend, Cat#103114, clone 30-F11),PE anti-mouse CD40 antibody (Biolegend, Cat#124610, clone 3/23), PerCP/Cyanine5.5 anti-mouse CD80 antibody (Biolegend, Cat#104722, clone 1610A1),PE/Cyanine7 anti-mouse CD86 antibody (Biolegend, Cat#105014, clone GL-1),PE anti-human CD16 antibody(Biolegend, Cat#980102, clone 3G8), PE/Cyanine7 anti-human CD66b antibody(Biolegend, Cat#305116, clone G10F5).

focus-forming assays.
anti-Zika virus antibody was produced and purify from hybridoma D1-4G2-4-15 (ATCC HB-112), Mouse IgG (H+L) Secondary Antibody (ThermoFisher, cat#31430).

Validation: The validation of the antibodies can be found in data sheet in the relevant manufactures' websites that described in the manuscript.

Eukaryotic cell lines

Policy information about cell lines and Sex and Gender in Research

Cell line source(s): Vero cells (ATCC CCL-81)

Authentication: cell were confirmed for viability and morphology before used.

Mycoplasma contamination: negative for mycoplasma

Commonly misidentified lines (See ICLAC register): No commonly misidentified cell lines are in this study.

Animals and other research organisms

Policy information about studies involving animals; ARRIVE guidelines recommended for reporting animal research, and Sex and Gender in Research

Laboratory animals: Female C57BL/6 mice or AG129 mice (6-8weeks of age).

Wild animals: There were no wild animals used in this study.

Reporting on sex: All mice involved in this study were females.

Field-collected samples: This study did not involve samples collected on the field.

Ethics oversight: All animal experimental protocols were approved by the Institutional Animal Care and Use Committee (IACUC) of the National Health Research Institutes.

Note that full information on the approval of the study protocol must also be provided in the manuscript.

Flow Cytometry

Plots

Confirm that:

☒ The axis labels state the marker and fluorochrome used (e.g. CD4-FITC).

☒ The axis scales are clearly visible. Include numbers along axes only for bottom left plot of group (a 'group' is an analysis of identical markers).

☒ All plots are contour plots with outliers or pseudocolor plots.

☒ A numerical value for number of cells or percentage (with statistics) is provided.

Methodology

Sample preparation: Neutrophil-mediated phagocytosis of Staphylococcus aureus

Sample preparation

Phagocytosis was measured with FITC-labeled Staphylococcus aureus strain ATCC 25923. Purified human neutrophils (determined as CD16+ CD66b+ cells) were added at a 10:1 bacteria/cell ratio and incubated for the indicated time at 37 °C with a 750 rpm shaker. The reaction was stopped with ice-cold paraformaldehyde (3.7%). After 2 washes with PBS, cell-associated fluorescent bacteria were analyzed by flow cytometry. Phagocytosis was determined as the relative geometric mean fluorescence(FITC) intensity of cells with fluorescent bacteria.

Activation of bone marrow-derived dendritic cell
The femurs and tibiae of C57BL/6 mice were obtained, and the bone marrow cells were dispersed by vigorous pipetting. The cells were then treated with lysis buffer to remove red blood cells, and the isolated bone marrow cells were resuspended (5× 10^5 cells/mL) in RPMI-1640 supplemented with 10% (v/v) heat-inactivated fetal bovine serum, penicillin/streptomycin (50 units/mL), l-glutamine (2 mM), HEPES (20 mM), and β-mercaptoethanol (50 μM) at 37 °C under 5% CO2. On days 0 and 3, granulocyte macrophage colony stimulating factor (200 units/mL) was added to the cultures. Cultured cells were harvested on day 6. One-mL aliquots of suspended BMDCs (1×10^6 cells/mL) were seeded into 24-well plates and stimulated with rF or rLF (5 μg/mL). Cells were harvested for surface marker staining with APC/Cyanine7 anti-CD11c (N418), FITC anti-MHCII (M5/114.15.2), PE anti-CD40 (3/23), PerCP/Cyanine5.5 anti-CD80 (16-10A1) and PE/Cyanine7 anti-CD86 (GL-1) monoclonal antibodies after 20 h of stimulation. Staining antibodies were obtained from Biolegend. The expression of surface markers was analyzed by flow cytometry (AttuneTM NxT, Thermo Fisher Scientific) on gated CD11c+ MHCII+ cell populations.

Analysis of antigen uptake by NALT dendritic cells
rOVA was labeled with an Alexa Fluor 700 labeling kit (Abcam, Cambridge, UK). Groups of C57BL/6 mice (6–8 weeks of age) were intranasally administered 30 μg of Alexa Fluor 700-labeled rOVA or Alexa Fluor 700-labeled rOVA adjuvanted with 10μg of rF or rLF. At 18 hours after administration, a single NALT cell suspension (pooled from 3 mice/group) was collected. The Zombie Violet™ Fixable viability kit (Biolegend, San Diego, CA) was used to evaluate the viability of NALT cells by flow cytometry. Lymphocytes were distinguished by staining with PE/Cyanine7-conjugated anti-CD45 antibody (30-F11). Dendritic cells were distinguished by staining with Alex Fluor488-conjugated anti-MHCII antibody (M5/114.15.2) and PE-conjugated anti-CD11c antibody (N418). Staining antibodies were obtained from Biolegend. The frequency of the antigen-positive (Alexa Fluor 700+) DCs in draining NALT was analyzed by flow cytometry.

Instrument

Attune NxT Flow Cytometer (Thermo Fisher Scientific)

Software

The data were acquired using AttuneTM NxT software and analyzed using FlowJo 10.8.1 software.

Cell population abundance

In total, at least 5 million events were acquired for subsequent analysis to identify rare populations

Gating strategy

All samples were initially gated using forward scatter and side scatter to identify events corresponding to cells, and then using side scatter height vs. area to enrich for single cells, next alive cells were selected by negativity for viability dye. The follow gaiting steps are presented in principal and supplementary figures.

☒ Tick this box to confirm that a figure exemplifying the gating strategy is provided in the Supplementary Information.
